# Split-Hand Syndrome in Amyotrophic Lateral Sclerosis: Differences in Dysfunction of the FDI and ADM Spinal Motoneurons

**DOI:** 10.3389/fnins.2019.00371

**Published:** 2019-05-08

**Authors:** Zhi-Li Wang, Liying Cui, Mingsheng Liu, Kang Zhang, Shuangwu Liu, Qingyun Ding

**Affiliations:** ^1^Department of Neurology, Peking Union Medical College Hospital, Chinese Academy of Medical Sciences, Beijing, China; ^2^Neurosciences Center, Chinese Academy of Medical Sciences, Beijing, China

**Keywords:** amyotrophic lateral sclerosis, F-wave, motor neuron, split-hand, first dorsal interosseous muscle, motor neuron disease

## Abstract

The F-wave test allows for the non-invasive assessment of spinal motoneuron excitability. We investigated the difference in spinal motoneuron dysfunction between the first dorsal interosseous (FDI) and abductor digit minimi (ADM) muscles by investigating F-waves and to assess the contribution of spinal mechanisms to split-hand syndrome in patients with amyotrophic lateral sclerosis (ALS). Sixty-five consecutive ALS patients and twenty age- and gender-matched healthy controls (HCs) were enrolled. Motor nerve conduction studies and F-waves were performed bilaterally on median and ulnar nerves in all subjects. HCs revealed prominently longer F-wave latencies, lower chronodispersion, mean F-wave amplitude, and mean and maximal F/M amplitude ratio (*P* < 0.001) in the FDI compared to the ADM. However, no significant differences in almost all F-wave parameters between the FDI and ADM were observed in ALS patients with affected hands except the minimal and mean F-wave latency. These data suggest that excitability is greatly changed in the spinal motoneurons innervating the FDI. Furthermore, the mean F-wave amplitude (*r* = 0.454, *P* = 0.002) of the FDI was significantly correlated with the FDI/ADM CMAP amplitude ratio in ALS patients with affected hands but not of the ADM. Our findings suggested that the dysfunction of spinal motoneurons between the FDI and ADM was different in ALS, and spinal motoneuron dysfunction was associated with development of the split-hand phenomenon.

## Introduction

Amyotrophic lateral sclerosis (ALS) is a rapidly progressive neurodegenerative disorder involving both upper and lower motor neurons (LMNs) and is often characterized by muscle weakness and atrophy, especially the small hand muscles. Dissociated atrophy of intrinsic hand muscles as an early and specific clinical feature of ALS, termed the split-hand sign, refers to preferential weakness and wasting of the abductor pollicis brevis (APB) and first dorsal interosseous (FDI) muscles with relative sparing of the abductor digit minimi (ADM) (Kuwabara et al., 1999, 2008; Wilbourn, 2000; Eisen and Kuwabara, 2012; Eisen et al., 2017). Menon et al. (2014b) reported that the split-hand sign was often evident in 62% of patients at the time of visiting and in 95% at follow-up. Although the thenar complex muscles (APB/FDI) and hypothenar muscles (ADM) constituting the split-hand are innervated through the same spinal segments (C8 and T1), the FDI and ADM, which are differentially affected, share ulnar innervation (Weber et al., 2000; Kuwabara et al., 2008; Eisen and Kuwabara, 2012). Corticomotoneuronal input and spinal/peripheral mechanisms have been suggested to be involved (Weber et al., 2000; Bae et al., 2009; Eisen and Kuwabara, 2012; Shibuya et al., 2013; Menon et al., 2014b; Eisen et al., 2017), and cortical dysfunction is considered as the likely pathophysiological mechanism underlying the split-hand phenomenon, while axonal dysfunction may appear as a downstream process (Menon et al., 2014a,b). The spinal mechanisms underlying the development of split-hand in ALS remain controversial. For example, Cengiz et al. (2018) reported no significant difference in cutaneous silent period measurements between the ADM and FDI, suggesting no role of spinal cord excitability changes in split-hand syndrome. However, Wilbourn (2000) reported the finding of split-hand in ALS in 1992, as well as in other diseases with only LMN dysfunction, and suggested that the lesion responsible for the ALS split-hand was at the level of the cervical anterior horn cell (Schelhaas et al., 2003). Further, Fang et al. (2016) found differences between the dysfunction of spinal motoneurons innervating the APB and the ADM in ALS. Thus, in the present study, we examined the hypothesis that spinal mechanisms contribute to split-hand in ALS.

The F-wave is a late response that reflects antidromic activation of motoneurons. Previous studies have shown that F-waves were not only used to assess changes in the excitability of spinal motoneurons (Espiritu et al., 2003; Lin and Floeter, 2004; Argyriou et al., 2006) but also as a probe to determine the activity of the motor cortex (Mercuri et al., 1996; Rivner, 2008). A direct comparison of the F-wave variables of the FDI and ADM innervated by the same nerve and the same spinal segments may provide more valuable information on the excitability changes of the spinal motoneuron pool and shed light on the complex mechanisms of split-hands. To date, the characteristics of multiple F-wave variables in the FDI have not been assessed in healthy subjects and ALS in previous studies. Therefore, the aims of this study were to (1) identify the characteristics of F-waves of the FDI, and (2) to ascertain the difference in spinal motoneuron dysfunction between the FDI and ADM in patients with ALS and HCs and to clarify the spinal pathophysiology of split-hand.

## Materials and Methods

### Subjects

Sixty-five consecutive patients diagnosed as having definite, probable and laboratory-supported probable sporadic ALS according to the revised El Escorial criteria were included in this study. All patients were recruited at the department of neurology in Peking Union Medical College Hospital between December 2017 and November 2018. Patients with ALS complicated by diabetic neuropathy, alcohol abuse, carpal tunnel syndrome, cervical myelopathy, and other neurological disorders were excluded. Control data were obtained from 20 age- and gender-matched healthy volunteers, whose nerve condition studies were normal. In each patient the muscle strength was assessed using the Medical Research Council (MRC) score, and a total MRC score was calculated for the following muscle groups assessed bilaterally: shoulder abduction, elbow flexion, elbow extension, wrist dorsiflexion, finger abduction, thumb abduction, hip flexion, knee extension, and ankle dorsiflexion (Menon et al., 2014b). The maximum possible total MRC score was 90. The clinical status of each patient was evaluated with the ALS Functional Rating Scale-Revised (ALSFRS-R) and upper motor neuron (UMN) score, as previous studies described (Cedarbaum et al., 1999; Grapperon et al., 2014). Two groups were established from the ALS patients, an affected hand group with wasting and weakness in the intrinsic hand muscles, where the data from the more affected hands were analyzed (45 patients), and an unaffected hand group, where the data for bilateral hands were analyzed in this group (20 patients). The hand was considered to be unaffected if the intrinsic hand muscles contained APB, FDI and ADM of normal strength; no wasting or weakness; and the nerve conduction studies (NCSs) were within normal limits. The hands of the healthy controls (HCs) were analyzed bilaterally. To estimate the influence of UMN involvement in the split-hand phenomenon, two subgroups were formed from ALS patients in the affected hand group, designated as the P group (pyramidal signs) and the NP group (no pyramidal signs). A more conservative but robust criterion for UMN lesion was used in the present study, requiring both increased tendon reflexes and positive Hoffman’s sign in defining the presence of pyramidal lesion in the arm (de Carvalho et al., 2002). The study was approved by the Peking Union Medical College Hospital Clinical Research Ethics Committee (Beijing, China), and all participants provided signed informed consent.

### Nerve Conduction Studies

All patients underwent routine NCSs and electromyography (EMG) using an EMG machine (Medtronic-Dantec Electronics, Skovlunde, Denmark). A peak-to-peak amplitude of maximal compound muscle action potential (CMAP) was elicited by using supramaximal (120%) surface stimulation of the median and ulnar nerves at the wrist and recorded from the APB, FDI and ADM muscles according to previously described standard methods (Stimulus duration: 0.1 ms; Filter setting: 20 Hz–10 kHz Gain: 200 μV/division; Sweep speed: 5 ms/division). Specifically, for FDI recording, the active electrode (G1) was placed on its belly and the reference electrode (G2) at the medial aspect of the proximal interphalangeal joint of the index finger (Kuwabara et al., 2008). The distance between the cathode and active (G1) recording electrodes for ADM muscles was 6.5 cm, while the distance between the cathode and active (G1) electrode for the FDI muscle was 8–10 cm. There was no evidence of conduction block or M response temporal dispersion in ALS patients. The skin temperature was maintained above 32°C. The following parameters were obtained: distal motor latency (DML), motor conduction velocity (MCV), CMAP amplitude (peak-to-peak), and the FDI/ADM CMAP amplitude ratio.

### F-Wave Studies

The F-waves of ulnar nerves were recorded with surface electrodes attached to the skin over the FDI and ADM muscles, the same position as in motor nerve conduction studies (de Carvalho et al., 2002; Kim, 2011). One hundred consecutive supramaximal (120%) percutaneous stimuli were delivered to the ulnar nerve at the wrist at a frequency of 1 Hz with the cathode proximal to the anode (Filters setting: 20 Hz–3 kHz; amplifier gain: 200 μV/division). A peak-to-peak deflection from baseline of at least 40 μV was accepted as an F-wave (Peioglou-Harmoussi et al., 1985). The following F-wave variables were measured in the FDI and ADM: the minimum, mean and maximum latency corrected according to the subject’s height (FLmin/H, FLmax/H, FLmean/H) (ms/m); chronodispersion; persistence; mean and maximum F-wave amplitude (peak-to-peak); mean and maximum F/M amplitude ratio (average or maximum peak-to-peak amplitude of F-waves expressed as a percentage of maximum distal CMAP amplitude); and the number of repeater F-waves. The repeater F-waves were identified as having the same shape, latency, and amplitude, and were calculated by the following indices as described by Chroni et al. (Chroni et al., 2012): index repeating neuron (index RN) (number of repeating neuron/ number of traces with different F-wave shapes in a series of 100 stimuli × 100), and index repeater F-waves (index Freps) (total number of F-wave repeaters/total number of traces with F-waves in the same nerve × 100). Due to the nature of the applied F-wave technique, which requires recording of a significant number of F-waves, we only examined the FDI and ADM muscles with strength of MRC of 2 or higher. And nerves without F-waves or the CAMP amplitude ≤ 2.0 mV were excluded from our analysis.

### Statistical Analysis

All analyses were performed using SPSS for windows version 24.0 (SPSS, IBM, Chicago, IL, United States). Normality was checked by the Shapiro–Wilk test. Normally distributed data are expressed as the mean ± SD and were compared using one-way ANOVA and the Student-Newman-Keuls (SNK) test. Mean values of measured variables between the FDI and ADM within the same group were compared using Student’s *t*-test. Non-normally distributed data are expressed as the medians (IQR) and were compared using the Kruskal–Wallis *H*-test. Once the null hypothesis was rejected, pairwise comparisons of the groups were tested using the Mann–Whitney *U*-test and Bonferroni correction with a significance level of *P* < 0.017. The relationship between the F-wave parameters and FDI/ADM CAMP amplitude ratio was assessed using Pearson’s correlation and Spearman’s rank correlation test. For comparison of the frequency distribution of categorical variables (gender and disease onset), the χ^2^ test was used. The level of statistical significance was established at *P* < 0.05.

## Results

The clinical profiles of the ALS patients and HCs are presented in Table 1. Among the ALS groups, all patients studied herein had a clinically predominant LMN syndrome and none had a pure UMN syndrome. The total MRC scores were higher in the unaffected hand group than the affected hand group. Disease duration, UMN score and ALSFRS-R were not significantly different between the affected hand and unaffected hand groups. The age at examination, gender ratio, and height were comparable between patients and controls.

**Table 1:** Clinical profile of participants.

Parameters	Affected hand (A, *n* = 45)	Unaffected hand (B, *n* = 20)	HCs (C, *n* = 20)	*P*-value		
				A vs. C	B vs. C	A vs. B
Age (year)	53.42 ± 8.82 (34–66)	51.20 ± 9.71 (35–69)	52.4 ± 9.13 (39–73)	>0.05	>0.05	>0.05
Gender (male:female)	25:20	7:13	11:9	>0.05	>0.05	>0.05
Height (cm)	165.84 ± 8.57	163.85 ± 7.56	166.3 ± 8.18	>0.05	>0.05	>0.05
Disease duration (months)	14.73 ± 8.89 (3–45)	11.70 ± 6.97 (4–27)	NA	NA	NA	0.158
Disease onset (bulbar: upper limbs: lower limbs)	9:28:8	9:3:8	NA	NA	NA	0.002
Total MRC scores	73.44 ± 10.27 (43–88)	83.35 ± 6.78 (67–90)	NA	NA	NA	<0.001
UMN scores	39.24 ± 14.03 (4–64)	32.10 ± 12.87 (5–54)	NA	NA	NA	0.057
ALSFRS-R	40.44 ± 4.18 (28–47)	42.30 ± 2.76 (36–46)	NA	NA	NA	0.099

*HCs, healthy controls; MRC, Medical Research Council; UMN, upper motor neuron; ALSFRS-R, amyotrophic lateral sclerosis functional rating scale-revised; NA, not applicable.*

Table 2 summarizes the overall comparisons between motor conduction values obtained from the ulnar nerves (FDI and ADM) of both patients with ALS and HCs. In HCs, the mean CMAP amplitude in FDI was greater than that in ADM, and the mean FDI/ADM CMAP amplitude ratio was calculated as 1.38. A significant reduction of FDI/ADM CMAP amplitude ratio (0.9 ± 0.3) was observed in the affected hand group compared with HCs, confirming that the split-hand phenomenon was evident in the present ALS patients (Kuwabara et al., 2008).

**Table 2:** Results of nerve conduction studies and split-hand.

Parameters	Affected hand (A, *n* = 45)	Unaffected hand (B, *n* = 40)	HCs (C, *n* = 40)	*P*-value		
				A vs. C	B vs. C	A vs. B
**DML (ms)**						
FDI	3.60 ± 0.40	3.45 ± 0.38	3.42 ± 0.32	**0.005**	0.519	0.089
ADM	2.47 ± 0.45	2.20 ± 0.26	2.19 ± 0.25	**0.001**	0.784	**0.002**
**CMAP amplitude (mV)**						
FDI	7.05 ± 4.31	17.91 ± 4.73	17.78 ± 3.77	<**0.001**	0.825	<**0.001**
ADM	7.49 ± 3.41	13.31 ± 2.80	14.62 ± 2.85	<**0.001**	0.027	<**0.001**
**FDI/ADM CMAP amplitude ratio**	0.90 ± 0.30	1.42 ± 0.28	1.38 ± 0.21	<**0.001**	0.593	<**0.001**
**MCV (m/s)**						
FDI	56.99 ± 4.53	60.11 ± 1.94	60.18 ± 1.45	<**0.001**	0.159	<**0.001**
ADM	55.89 ± 3.66	60.12 ± 1.22	60.20 ± 1.46	<**0.001**	0.102	<**0.001**

*DML, distal motor latency; FDI, first dorsal interosseous; ADM, abductor digit minimi; CMAP, compound muscle action potential; MCV, motor conduction velocity; HCs, healthy controls. All data are expressed as the mean ± SD. Values with significant differences printed in bold characters.*

**FIGURE 1:**
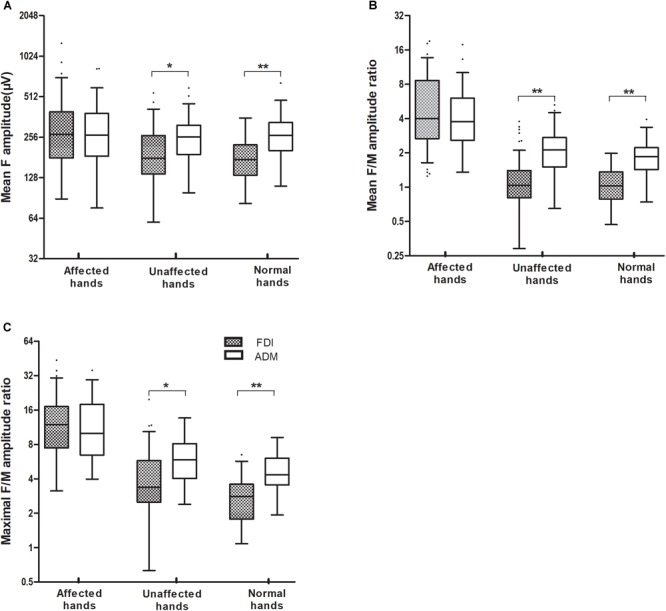
The mean F-wave amplitude **(A)**, and the mean and maximal F/M amplitude ratios **(B,C)**, recorded over the FDI and ADM muscles were significantly increased in ALS patients with affected hands compared with those with unaffected hands. When the FDI and ADM were compared in HCs and the unaffected hand group, the FDI showed a noticeably lower mean F-wave amplitude, and a lower mean and maximal F/M amplitude ratios, than for the ADM. By contrast, there were no differences in F-wave measurements between the FDI and ADM in the affected hand group. ^∗∗^*P* < 0.001; ^∗^*P* < 0.05. The numerical data on the Y-axis were logarithmically transformed (Log2).

The results of F-wave variables are displayed in Table 3. When the FDI and ADM were compared in HCs, the FDI showed noticeably longer F-wave latencies and lower chronodispersion, mean F-wave amplitude, and mean and maximal F/M amplitude ratios than the ADM. This trend was similar in the unaffected hand group (Figure 1A–C). In contrast, no differences between the FDI and ADM for F-wave measurements were observed in the affected hand group except the FLmin/H and FLmean/H. Concerning the F-wave variables in the unaffected hand group, the F-wave latencies, persistence, chronodispersion of the FDI and ADM and index RN, and index Freps of the FDI were significantly changed compared to controls, along with the relative normal mean F-wave amplitude, mean and maximal F/M amplitude ratios of the FDI and ADM and index RN and index Freps of the FDI (column B vs. C). Table 4 shows the comparison of F-wave parameters in ALS patients with pronounced split-hands between subgroups of those with (P) and without (NP) pyramidal signs. No difference was observed between the P and NP groups. Additionally, the difference between the FDI and ADM was not significant in both groups.

**Table 3:** Results of F-wave variables in the ALS patients and the healthy controls.

Parameters	Affected hand (A, *n* = 45)	Unaffected hand (B, *n* = 40)	HCs (C, *n* = 40)	*P*-value		
				A vs. C	B vs. C	A vs. B
**Minimal F latency (ms/m)**						
FDI	16.37 ± 0.95^∗∗^	15.84 ± 0.71^∗∗^	15.13 ± 0.46^∗∗^	<**0.001**	<**0.001**	0.022
ADM	15.62 ± 0.92^∗∗^	14.93 ± 0.79^∗∗^	14.50 ± 0.47^∗∗^	<**0.001**	**0.016**	<**0.001**
**Maximal F latency (ms/m)**						
FDI	18.93 ± 1.78	17.70 ± 0.92^∗^	16.74 ± 0.57^∗∗^	<**0.001**	<**0.001**	<**0.001**
ADM	18.59 ± 1.70	16.99 ± 0.89^∗^	15.13 ± 0.46^∗∗^	<**0.001**	<**0.001**	<**0.001**
**Mean F latency (ms/m)**						
FDI	17.41 ± 1.15^∗^	16.53 ± 0.72^∗∗^	15.82 ± 0.54^∗∗^	<**0.001**	<**0.001**	<**0.001**
ADM	16.80 ± 1.03^∗^	15.81 ± 0.84^∗∗^	15.17 ± 0.52^∗∗^	<**0.001**	<**0.001**	<**0.001**
**F-wave chronodispersion (ms)**						
FDI	4.22 ± 2.27	3.04 ± 0.95^∗^	2.49 ± 0.55^∗^	<**0.001**	**0.001**	**0.004**
ADM	4.91 ± 2.31	3.34 ± 0.70^∗^	2.88 ± 0.66^∗^	<**0.001**	**0.003**	<**0.001**
**F-wave persistence (%)**						
FDI	62 (41)	96.5 (9.25)^∗^	100 (0.75)	<**0.001**	<**0.001**	<**0.001**
ADM	71 (54.5)	99 (1)^∗^	100 (0)	<**0.001**	**0.002**	<**0.001**
**Mean F-wave amplitude (μV)**						
FDI	269 (216.5)	178.5 (127.25)^∗^	174.5 (90.75)^∗∗^	**0.002**	0.648	**0.015**
ADM	266 (199.5)	257.5 (124)^∗^	264.5 (126.5)^∗∗^	>0.05	>0.05	>0.05
**Mean F/M amplitude ratio (%)**						
FDI	3.99 (5.96)	1.04 (0.6)^∗∗^	1.03 (0.57)^∗∗^	<**0.001**	0.950	<**0.001**
ADM	3.76 (3.47)	2.13 (1.23)^∗∗^	1.85 (0.80)^∗∗^	<**0.001**	0.258	<**0.001**
**Maximal F/M amplitude ratio (%)**						
FDI	11.97 (9.72)	3.37 (3.28)^∗^	2.80 (1.82)^∗∗^	<**0.001**	0.020	<**0.001**
ADM	10.01 (11.51)	5.86 (4.09)^∗^	4.33 (2.52)^∗∗^	<**0.001**	0.017	<**0.001**
**Index RN (%)**						
FDI	16.67 (23.07)	1.62 (3.23)^∗∗^	0 (0)	<**0.001**	<**0.001**	<**0.001**
ADM	18.18 (29.81)	0 (1.79)^∗∗^	0 (0)	<**0.001**	0.017	<**0.001**
**Index Freps (%)**						
FDI	55.81 (51.57)	4.12 (13.33)^∗∗^	0 (0)	<**0.001**	<**0.001**	<**0.001**
ADM	50 (59.12)	0 (3.77)^∗∗^	0 (0)	<**0.001**	0.017	<**0.001**

*HCs, healthy controls; FDI, first dorsal interosseous; ADM, abductor digit minimi. Normally distributed data are expressed as the mean ± SD, and non-normally distributed data are expressed as the medians (IQR). Values with significant differences printed in bold characters. ^∗∗^*P* <0.001, ^∗^*P* <0.05, between the FDI and the ADM in each group. For comparisons of F-wave variables among affected hand group, unaffected hand and healthy control group, Bonferroni correction with a significance level of *P* < 0.017.*

**Table 4:** Comparison of F-wave parameters in ALS patients with split-hands between subgroups of those with (P) and without (NP) pyramidal signs.

Parameters	P group (*n* = 21)	NP group (*n* = 24)	*P*-value
**F-wave persistence (%)**			
FDI	62 (40)	59 (42.75)	0.637
ADM	84 (50)	52.5 (60)	0.255
**Mean F-wave amplitude (μV)**			
FDI	219 (180)	310.5 (286)	0.481
ADM	209 (162)	326.5 (181.25)	0.062
**Mean F/M amplitude ratio (%)**			
FDI	3.47 (3.01)	5.03 (7.39)	0.387
ADM	2.89 (1.74)	4.26 (3.41)	0.055
**Maximal F/M amplitude ratio (%)**			
FDI	11 (8.97)	15.2 (18.11)	0.106
ADM	9.09 (7.65)	13.47 (12.10)	0.116

*FDI, first dorsal interosseous; ADM, abductor digit minimi; P, with pyramidal signs; NP, without pyramidal signs; Non-normally distributed data are expressed as the medians (IQR).*

The results of the correlation analysis conducted between the F-wave parameters and FDI/ADM CMAP amplitude ratio displayed in Supplementary Table S1. Combining these parameters, it was evident that the F-wave amplitude (*r* = 0.454, *P* = 0.002) of the FDI was significantly correlated with the FDI/ADM CMAP amplitude ratio in the affected hand group, but not with the ADM. There was no significant correlation between other F-wave variables in both the ADM and FDI and the FDI/ADM CMAP amplitude ratio in the affected hand group of ALS patients. No significant relation was observed between the F-wave variables and FDI/ADM CMAP amplitude ratios in the unaffected hand group and HCs.

## Discussion

### Results Related to the Changes in ALS

Our NCSs variables of the FDI and ADM and F-wave values of the ADM in HCs and patients with ALS showed a close resemblance to the previous findings (Peioglou-Harmoussi et al., 1985; Kuwabara et al., 2008; Buschbacher et al., 2015; Fang et al., 2016). In the present study, ALS patients with an unaffected hand did not show significant changes in DML, CMAP amplitude, or MCV recorded over the FDI and ADM in contrast with HCs. While a significantly decreased CMAP amplitude was associated with increased DML and slowed MCV in both the FDI and ADM were observed in our patients with affected hands. These findings are compatible with the chronic denervation/reinnervation process, and are associated with the pathophysiological changes in ALS (de Carvalho et al., 2002; Argyriou et al., 2006).

We used F-waves as an indicator of dysfunction of spinal motoneurons. The F-wave amplitudes are related to the excitability of spinal motoneurons and axonal compensatory reinnervation (Argyriou et al., 2006; Hachisuka et al., 2015). Specifically, the F/M amplitude ratio was used as a quantified index of the proportion of the motoneuron pool, as this measure is minimally influenced by muscle wasting (Argyriou et al., 2006). In ALS patients, the mean F-wave amplitude, and mean and maximal F/M amplitude ratios, were increased in the FDI and ADM. Similar findings were reported and inferred that both anterior horn cell hyperexcitability (Argyriou et al., 2006) and the formation of large post-reinnervation motor units due to LMN dysfunction (Drory et al., 2001) are important factors. In the present study, ALS patients also showed reduced F-wave persistence and increased repeater F-waves. Similar changes were reported in post-polio syndrome (PPS) (Hachisuka et al., 2015). Both F-wave persistence and repeater-F waves are influenced by the number of functional LMNs and motoneuron excitability. A low F-wave persistence indicates loss of function of LMNs and decreased excitability of the motoneuron pool (de Carvalho et al., 2002; Argyriou et al., 2006; Rivner, 2008). With respect to the mechanism of production of repeater F-waves in PPS, it was proposed that loss of motoneurons or decreased excitability of some anterior horn cells caused the remaining anterior horn cells with increased excitability to produce more frequent repeated backfiring (Hachisuka et al., 2015). This underlying pathophysiology in PPS may also explain the increased repeater F-waves in ALS (Chroni et al., 2012; Hachisuka et al., 2015). In addition, the F-wave latencies and chronodispersion were markedly prolonged in our ALS group. However, the F-wave latencies commonly thought to be influenced by height and preferential loss of fast-conduction neurons and the chronodispersion represent the conduction velocity of the motor neurons recruited, which are valuable markers of the conduction properties of motor axons (Fisher, 1998; Espiritu et al., 2003; Rivner, 2008). Their prolongation may be related to axonal degeneration, demyelination secondary to proximal axonal swellings or loss of fast conducting fibers (Argyriou et al., 2006; Hachisuka et al., 2015). As such, analyzing F-waves, especially the amplitude, mean and maximal F/M amplitude ratios, persistence, and the repeater F-waves, may provide an indicator of changes in spinal motoneuron pool excitability (Espiritu et al., 2003; Lin and Floeter, 2004). Intriguingly, we note that F-wave latencies and chronodispersion prolongation, persistence decline and repeater F-waves increase progressively early in patients with unaffected hands, suggesting early dysfunction of motor axons and LMNs in ALS and that subtle subclinical alterations may be reliably assessed by F-wave test.

### Results Related to Split-Hand Syndrome

Our study shows a significantly decreased FDI/ADM CMAP amplitude ratio (<0.9) in ALS patients, and this finding reflects the split-hand phenomenon in ALS (Kuwabara et al., 2008; Menon et al., 2013) and presents evidence of LMN involvement. Further, there was a higher CMAP amplitude of the FDI and a lower amplitude of F-waves compared with the ADM in HCs, which has not been previously reported. The reduction in F-wave amplitude is often caused by damage to the LMNs and decreased motor neuron excitability (Taniguchi et al., 2008). The lower amplitude of F-waves, and the lower mean and maximal F/M amplitude ratios in the FDI compared with the ADM of HCs, is likely caused by physiological differences in the excitability of their motoneuron pools, and may relate to central impulses in favor of inhibition in the FDI (Menon et al., 2014c). Similar physiological differences, including lower mean F-wave amplitude, and lower mean and maximal F/M amplitude ratios, in the FDI, compared with the ADM, were also observed in ALS patients with unaffected hands. In addition, compared with the ADM, lower F-wave persistence and increased repeater F-waves were observed in the FDI in the unaffected hands group. These findings suggest a greater degree of spinal motoneuron hypoexcitability and loss of function of LMNs in the FDI. However, patterns of F-wave measurements changes in this study, especially those parameters detecting excitability of the spinal motoneuron pool (F-wave persistence, amplitudes, mean and maximal F/M amplitude ratio and repeater F-waves), were similar in FDI and ADM in ALS patients with affected hands. The absence of differences in F-wave variables between the FDI and ADM in ALS may imply a significantly enhanced excitability of spinal motoneurons innervating the FDI. We also found a significant correlation between F-wave amplitude in the FDI with the FDI/ADM CMAP amplitude ratio, but not with the ADM, suggesting that the different changes in spinal motoneuron excitability between the FDI and ADM were associated with development of the split-hand phenomenon in ALS.

At the segment spinal motoneuron level, the excitability of the motoneuron pool may be affected by the excitatory and inhibitory central nervous system (Mastaglia and Carroll, 1985; de Carvalho et al., 2002). To further clarify the impact of UMN activity drive on the excitability of the anterior horn cells in ALS patients with split-hand, we examined the F-wave parameters in our subgroup, including persistence, amplitude, and the F/M amplitude ratio, which presumptively are influenced by the corticospinal tract and cortical activity (Lin and Floeter, 2004; Rivner, 2008; Hara et al., 2010). However, we found no differences in F-waves between the P and the NP subgroups or between the FDI and ADM subgroups. The differences in segmental motoneuron excitability were not closely correlated to UMN involvement in our study. Thus, we suspect that the pathophysiology of the split-hand may also have spinal mechanisms.

Our study has some limitations. This was exploratory research with a small sample size. Thus, more patients and follow-up studies are required to confirm our findings on spinal motoneuron excitability associated with split-hand syndrome in ALS. Because of the stimulation of the ulnar at the wrist, the distance from the stimulus site to the target muscle is considerably longer for the FDI than for the ADM. The comparison of F-wave latencies in the FDI and ADM may be of less value. Owing to the predominant involvement of LMN in ALS, signs of pyramidal lesions may be difficult to detect. Moreover, we defined pyramidal lesions in the upper limbs requiring both increased tendon reflexes in the arm and Hoffman’s sign, which may lack sensitivity. So, subclinical or possible involvement of UMN cannot be excluded in the NP group, and because of the relative small sample size, the results of F-wave parameters between the P and NP subgroup need to be verified in a larger population of ALS patients. Moreover, F-waves do not allow for accurate measurement of changes in UMN excitability influenced on spinal motoneurons, and a reliable method is needed for further studies. Combining transcranial magnetic stimulation (TMS) with the F-wave test investigates the UMN involvement and spinal motoneuron excitability at the same time and on the same patient groups could elucidate the pathophysiological basis of the split-hand in ALS.

In summary, the present study draws attention to a particular pattern of F-wave abnormalities in the FDI and ADM. Spinal motoneurons innervating the FDI have physiologically greater inhibitory modulation than the ADM, and in ALS, the enhanced excitability is more prominent in spinal motoneurons innervating the FDI that is consistent with the split-hand sign. Although cortical mechanisms could also be involved, we propose that spinal motoneurons dysfunction is associated with the development of the split-hand syndrome.

## Ethics Statement

This study was carried out in accordance with the recommendations of the Peking Union Medical College Hospital Clinical Research Ethics Committee (Beijing, China) with written informed consent from all subjects. All subjects gave written informed consent in accordance with the Declaration of Helsinki. The protocol was approved by the Peking Union Medical College Hospital Clinical Research Ethics Committee (Beijing, China).

## Author Contributions

Z-LW, ML, and LC designed the experiments and/or interpreted the data. Z-LW and QD performed the experiments and analyzed the data. ML, SL, and KZ contributed to reagents, materials, and analysis tools. Z-LW and LC drafted the manuscript.

## Conflict of Interest Statement

The authors declare that the research was conducted in the absence of any commercial or financial relationships that could be construed as a potential conflict of interest.
