# Supplementary material for: Split-Hand Syndrome in Amyotrophic Lateral Sclerosis: Differences in Dysfunction of the FDI and ADM Spinal Motoneurons
Source: Front Neurosci. 2019 May 8;13:371. doi: 10.3389/fnins.2019.00371 (PMC6517473; doi:10.3389/fnins.2019.00371)
Supplement: Supplementary file 1 [file Table_1.DOCX]

Supplementary Material

## Supplementary Table

**Table S1. Correlation analysis of F-wave variables with FDI/ADM CMAP amplitude ratio.**

| Parameters | Affected hand FDI/ADM ratio (A, n = 45) | | Unaffected hand FDI/ADM ratio (B, n = 40) | | HCs FDI/ADM ratio (C, n = 40) | |
| --- | --- | --- | --- | --- | --- | --- |
|  | r | *P* value | r | *P* value | r | *P* value |
| Minimal F latency (ms/m) |  |  |  |  |  |  |
| FDI | -0.149 | 0.329 | 0.126 | 0.438 | 0.158 | 0.331 |
| ADM | 0.056 | 0.713 | 0.132 | 0.415 | 0.021 | 0.898 |
| Maximal F latency (ms/m) |  |  |  |  |  |  |
| FDI | -0.115 | 0.452 | 0.153 | 0.347 | 0.059 | 0.715 |
| ADM | -0.036 | 0.812 | 0.104 | 0.521 | 0.158 | 0.331 |
| Mean F latency (ms/m) |  |  |  |  |  |  |
| FDI | -0.205 | 0.178 | 0.220 | 0.172 | 0.108 | 0.509 |
| ADM | -0.051 | 0.739 | 0.142 | 0.384 | 0.043 | 0.791 |
| F wave chronodispersion (ms) |  |  |  |  |  |  |
| FDI | -0.1880 | 0.236 | 0.050 | 0.761 | -0.168 | 0.300 |
| ADM | -0.087 | 0.570 | -0.228 | 0.157 | -0.249 | 0.121 |
| F-wave persistence (%) |  |  |  |  |  |  |
| FDI | 0.199 | 0.190 | 0.287 | 0.073 | 0.027 | 0.869 |
| ADM | 0.109 | 0.478 | 0.138 | 0.397 | 0.128 | 0.430 |
| Mean F-wave amplitude (µV) |  |  |  |  |  |  |
| FDI | 0.454 | **0.002** | 0.210 | 0.194 | 0.115 | 0.479 |
| ADM | -0.105 | 0.491 | 0.079 | 0.727 | 0.138 | 0.394 |
| Mean F/M amplitude ratio (%) |  |  |  |  |  |  |
| FDI | -0.273 | 0.090 | -0.057 | 0.726 | 0.095 | 0.559 |
| ADM | -0.253 | 0.094 | 0.227 | 0.259 | 0.364 | 0.052 |
| Maximal F/M amplitude ratio (%) |  |  |  |  |  |  |
| FDI | -0.217 | 0.152 | 0.173 | 0.286 | 0.202 | 0.211 |
| ADM | -0.317 | 0.084 | 0.257 | 0.109 | 0.269 | 0.093 |
| Index RN (%) |  |  |  |  |  |  |
| FDI | 0.110 | 0.470 | -0.067 | 0.681 | 0.111 | 0.494 |
| ADM | -0.245 | 0.104 | -0.025 | 0.879 | 0.027 | 0.871 |
| Index Freps (%) |  |  |  |  |  |  |
| FDI | 0.095 | 0.535 | -0.008 | 0.959 | 0.111 | 0.494 |
| ADM | -0.185 | 0.224 | -0.020 | 0.905 | 0.030 | 0.853 |

HCs, healthy controls; FDI, first dorsal interosseous; ADM, abductor digit minimi;

FDI/ADM ratio, FDI/ADM CAMP amplitude ratio; r, Correlation coefficient.
